# Attachment in close relationships and glycemic outcomes in children with type 1 diabetes

**DOI:** 10.1186/s13034-023-00672-1

**Published:** 2023-10-17

**Authors:** Simona Klemenčič, Jasna Klara Lipovšek, Anja Turin, Klemen Dovč, Nataša Bratina, Yael Shmueli-Goetz, Katarina Trebušak Podkrajšek, Barbka Repič Lampret, Barbara Jenko Bizjan, Sašo Karakatič, Tadej Battelino, Maja Drobnič Radobuljac

**Affiliations:** 1 Department of Pediatric Endocrinology, Diabetes and Metabolic Diseases, University Children’s Hospital Ljubljana, University Medical Centre Ljubljana, Bohoričeva Ulica 20, 1000 Ljubljana, Slovenia; 2 Centre for Mental Health, University Psychiatric Clinic Ljubljana, Ljubljana, Slovenia; 3 Faculty of Medicine, University of Ljubljana, Ljubljana, Slovenia; 4 Anna Freud National Centre for Children and Families and Psychoanalysis Unit, University College London, London, UK; 5 Clinical Institute of Special Laboratory Diagnostics, University Children’s Hospital, University Medical Centre Ljubljana, Ljubljana, Slovenia; 6 Institute of Informatics, Faculty of Electrical Engineering and Computer Science, University of Maribor, Maribor, Slovenia

**Keywords:** Attachment, Childhood and adolescence, Diabetes control, Time in range, Cortisol

## Abstract

**Background:**

Our aim was to determine whether child attachment to parents, parent attachment style, and morning cortisol levels were related to diabetes outcomes measured by average glycated hemoglobin (HbA1c), HbA1c variability over 4 years and time in range (TIR) in children with type 1 diabetes (T1D).

**Research design and methods:**

101 children with T1D and one of their parents were assessed at baseline for child attachment (Child Attachment Interview; CAI) and parent attachment (Relationship Structures Questionnaire; ECR-RS). Serum samples were collected for cortisol measurements before the interviews. HbA1c levels were measured during a 4-year follow-up period at regular 3-monthly visits, and data for TIR were exported from blood glucose measuring devices. Multivariate linear regression models were constructed to identify independent predictors of glycemic outcomes.

**Results:**

More girls than boys exhibited secure attachment to their mothers. The results of the regression models showed that securely attached girls (CAI) had higher average HbA1c than did insecurely attached girls (*B* = −0.64, *p* = 0.03). In boys, the more insecure the parent's attachment style, the worse the child's glycemic outcome: the higher the average Hb1Ac (*B* = 0.51,* p* = 0.005), the higher the HbA1c variability (*B* = 0.017, *p* = 0.011), and the lower the TIR (*B* = −8.543, *p* = 0.002).

**Conclusions:**

Attachment in close relationships is associated with glycemic outcomes in children with T1D, and we observed significant differences between sexes. A sex- and attachment-specific approach is recommended when treating children with less favorable glycemic outcomes. Special attention and tailored support should be offered to securely attached girls in transferring responsibility for diabetes care and at least to male children of insecurely attached parents to prevent suboptimal glycemic control. Further studies in larger samples and more daily cortisol measurements may help us better understand the links between stress response, attachment and T1D.

**Supplementary Information:**

The online version contains supplementary material available at 10.1186/s13034-023-00672-1.

## Background

Diabetes control in the early years after diagnosis of T1D may have an important impact on the long-term complications later associated with the individual's quality of life and diabetes-related burden. Several factors may impact diabetes control, for example, psychological stress may contribute via physiological mechanisms or lead to greater challenges associated with diabetes management [1].

Early experiences leading to the formation of an attachment between child and caregiver play a critical role in regulating individual responses to stress [2, 3]. According to Bowlby's attachment theory, individual attachment reflects dyadic interactions and is shaped in part by the behavior of adults important to the child (usually the parents or other primary caregivers). Over time, these repeated interactions become internalized and may determine biological and behavioral responses to stressful events [4]. Thus, based on early attachment experiences, mental representations are formed that are imprinted in the developing limbic and autonomic nervous systems and shape and guide the individual's behavior throughout development by guiding emotion regulation and the formation of subsequent relationships [5, 6]. Secure attachment is characterized by effective regulation of an individual's internal organization and a developed capacity for emotion regulation, whereas individuals with insecure attachment organization (preoccupied/anxious ambivalent, or dismissing/avoidant) exhibit difficulties in emotion regulation and self-organization [7].

Insecure attachment in adults has been shown to be associated with higher risk for psychopathology, chronic medical disorders such as diabetes, and poorer contribution to diabetes self-management [8–10]. In contrast, securely attached adult patients are more likely to respond to illness with appropriate resilience and trust medical professionals, whereas insecurely attached patients may exhibit distrust or co-dependence in relationships with medical professionals, deny illness, or avoid treatment [7].

Results from a recent study of adolescents with T1D showed that mothers' self-reported perceptions of more secure adolescents' attachment were associated with better glycemic control, whereas neither fathers' perceptions nor adolescents' reports showed a significant association with glycemic control [11]. In addition, several studies have reported the longitudinal stability of attachment styles and their transmission across generations [12], and thus one can predict an effect of parental attachment on their children's diabetes control.

In addition, the experience of early adverse events and higher psychological vulnerability of individuals put them at risk for a deregulated stress response [13]. In families with less adaptive conditions, insecure attachment in childhood and exposure to chronic stress may lead to altered subject's cortisol stress reactivity [14–16]. In addition, an association between an increase in cortisol stress response and early adversity has been found in adolescents with T1D [17]. Prolonged secretion of stress hormones, especially cortisol, could damage the hippocampus and the hypothalamic–pituitary–adrenal (HPA) axis, which regulate the release of corticosteroids. Because of the hyperreactive HPA axis, suboptimal compensation of serum cortisol levels results in the persistence of high cortisol levels even when the individual is no longer exposed to the stressor [18]. Persistent dysregulation of stress hormones can have deleterious effects on physiological, emotional, and behavioral processes in humans [19] and contribute to the development of metabolic or autoimmune diseases [20, 21]. In addition, dysregulation of the HPA axis has been found in children with T1D [22] and its influence on blood glucose levels in adults with type 2 diabetes [23].

HbA1c has become widely accepted as a measure of glycemic control, but it lacks information about daily glucose fluctuations and acute complications. Therefore, glycemic variability, including temporal variability of HbA1c [24] and time in the 70–180 mg/dl range (Time In Range, TIR), has recently been proposed to complement HbA1c [25, 26]. While HbA1c variability has been shown in both clinical trials and retrospective studies to be an accurate predictor that increases the risk of cardiovascular complications and mortality compared with HbA1c levels alone [24, 27, 28], TIR is a comprehensive measure of variability of blood glucose [25]. According to the international consensus report, TIR is best provided by continuous glucose monitoring (CGM), an intermittent, real-time measurement of glucose concentration in interstitial fluid with a body-mounted sensor that provides a near- continuous series of glucose concentration measurements and has been associated with improved glycemic outcomes [25, 29]. When data from CGM are not available, TIR can be calculated from frequent measurements of glucose concentration in capillary blood taken five to seven times daily using a blood glucose monitor (BGM). Although these data are sparse compared with data from CGM, the ability to assess the association of TIR, as shown previously, provides a fairly high degree of agreement between results based on CGM and BGM measurements [30].

Previous research has attempted to understand the relationship between secure attachment, stress response, and T1D in children, but results have been inconsistent. For example, using a self-assessment questionnaire, Costa-Cordella et al. [31] found a significant negative association between children's attachment to their parents and HbA1c levels in boys but not in girls. In girls, this study showed that stronger maternal attachment avoidance was associated with better metabolic control. The opposite was found by Shayeghian et al. [32], who found an association between higher HbA1c levels and more pronounced alexithymia and less communication with the mother (both elements of insecure attachment) in girls. Bizzy et al. [33] reported no significant differences in attachment to parents between children with high HbA1c levels and those with Hb1Ac levels below 7%, using the Child Attachment Interview in a sample of 31 children.

Our goal, therefore, was to address this gap in understanding the relationship between child attachment to parents, parental attachment styles, child stress reactivity, and glycemic outcomes in children and adolescents with T1D. We aimed to do this by including and prospectively following the entire Slovenian population of children and adolescents with T1D and using the most reliable assessment methods available. The recommendations derived from our findings would help to better understand how psychological and family factors influence T1D in children and adolescents and tailor appropriate interventions accordingly.

## Materials and methods

A prospective study of children and adolescents with T1D is presented. Each patient and one of his or her parents (caregivers) were enrolled in a larger cross-sectional study and later followed up prospectively. Some results of the original study, which found associations between attachment and risk of childhood onset of T1D, have already been published [34]. At baseline, data on child and parent attachment and children’s morning serum cortisol were collected. During a 4-year follow-up period, data on diabetes progression were collected at regular 3-month outpatient visits.

### Participants and procedures

All children with T1D and their parents were identified from the Slovenian National Diabetes Registry and invited to participate in the study. Child-parent pairs who responded were followed up in the prospective, single-arm study at the University Medical Centre Ljubljana. The main inclusion criteria were an age of 8–15 years (inclusive) and a clinical diagnosis of T1D for at least 1 year. Exclusion criteria included intellectual disability or/and active psychosis.

Recruitment began in July 2015 and was completed in December 2019. Initial screening included obtaining informed consent, collecting serum samples for measurement of cortisol levels, and assessing children's attachment. Parents completed the sociodemographic and attachment survey. Glycemic outcomes were collected at the regular 3-monthly visits.

The study was registered at www.clinicaltrials.gov (NCT02575001) and approved by the National Medical Ethics Committee of the Republic of Slovenia (#60/08/13). Participation in the study was voluntary and confidential, and all participants or their parents signed an informed consent form before participation.

### General measures

General demographic and family characteristics were assessed with a special questionnaire reported by parents [35]. Questions on early childhood development and diabetes management were added to the original questionnaire.

### Glycemic outcomes

HbA1c levels were determined on site by an immunochemical method using the Siemens DCA Vantage Analyzer (Siemens Healthcare, Erlangen, Germany).

HbA1c variability was calculated by a method similar to that reported by Forbes [27]. Absolute HbA1c differences between visits greater than or equal to 0.5 were considered clinically significant and counted for each participant. The result was multiplied by 100 and divided by the number of HbA1c measurements.

TIR was calculated by exporting raw data files from glucose measuring devices during regular clinical visits and analyzed with the statistical program R, package cgmanalysis [36]. At least eight days of CGM data with at least 200 data points per day in each 3-month period were required for inclusion. For participants without CGM, data from intermittent BGM measurements were alternatively considered. At least 10 days with at least five BGM data points per day in each 3-month interval were required for inclusion.

### Morning serum cortisol

Morning cortisol levels were measured in blood samples taken before food intake between 7:00 and 9:00 am. Blood samples were filled into tubes without additives and centrifuged within 2 h after blood collection. Serum cortisol was quantitatively measured using a chemiluminescent competitive immunoassay on the IMMULITE^®^ 2000 Systems Analyzer (Siemens).

### Child attachment interview

The Child Attachment Interview (CAI) is a narrative-based assessment of children's attachment to caregivers developed for subjects aged 8–15 years [37]. It is a direct interview in which children's internal working models are elicited via questions about current attachment relationships and experiences. The evaluation is done by analyzing the transcripts and video analysis of the behavior. In relation to the relationship with each attachment figure, a classification is made into main attachment groups [(1) two-way classification: secure or insecure; (2) three-way classification: secure, preoccupied, or dismissing; or (3) four-way classification: secure, preoccupied, dismissing, or disorganized] [37]. For all analyses, the two-way classification was used because we assumed that the sample size would be too small for statistical analyses in some subgroups of the three-way and four-way classifications. Similar decisions were made by other investigators [11, 31, 33]. The protocols were scored by three independent accredited coders, who demonstrated high inter-rater reliability as previously reported (conducted on 20 interviews for each pair of coders) [34].

### Relationship structures questionnaire

The Relationship Structures Questionnaire (ECR-RS) was used as a measure of parents' attachment patterns in close relationships [38]. This questionnaire measures attachment to four attachment figures (to each of the subject's parents, to a partner, and to a best friend) using the same nine questions, with overall attachment calculated as the mean of all relationship domains. Each relationship domain has two dimensions: attachment-related anxiety and attachment-related avoidance. Low scores on both dimensions indicate secure attachment. The questionnaire has good psychometric properties: two-dimensional internal structure validity and high to excellent reliability (Cronbach's alpha above 0.7 for different attachment figures and domains) [38].

### Data analysis

After testing for normal distribution with the Kolmogorov–Smirnov test, the Student’s *t-*test, Fischer exact test, Mann–Whitney test, and Pearson’s chi-square test in the statistical package SPSS 21.0. (IBM SPSS Statistics) were used to evaluate differences between groups. The Pearson or the Spearman correlation coefficient was used to assess the relationship between two variables. The significance level was set at *α* = 0.05 for the two-tailed hypothesis testing.

Multivariable linear regression in the R program was used to predict average HbA1c (Model 1), HbA1c variability (Model 2), and TIR (Model 3). In Model 1 and Model 2, the independent values were child's attachment to mother, parent's attachment style, child's sex, age, and morning cortisol level. In Model 3, the same independent variables were used plus the type of glucose monitoring (CGM or BGM). Parents' attachment style was measured with a two-dimensional concept capturing attachment anxiety and attachment avoidance. Because of the high correlation between the two dimensions, we created two versions of all tree regression models (a—including attachment anxiety, b—including attachment avoidance) to avoid multicollinearity.

Before analysis, all independent variables were centered. The creation of the linear regression model followed the stepwise backward procedure, where we started with the model with all main effect terms and all combinations between pair variables as interaction terms. Then, the term removal procedure was performed, where the higher order terms with the highest statistical significance (*p*-values) were removed from the model and the model was re-evaluated based on the calculation of the Akaike Information Criterion (AIC). This procedure of backward stepwise removal of terms continued as the AIC metric decreased, and the resulting model was the one with the lowest AIC at the end of the stepwise procedure [39]. Linear regression with two-tailed hypothesis testing and significance level *α* = 0.05 was used for the analysis.

In order to ensure the adequacy of our sample size for the three linear multiple regression models, we conducted sample size calculations using G*Power. The calculations were based on the following parameters: two-tailed tests, an effect size *f*^*2*^ of 0.15, an alpha level of 0.05, and a desired statistical power of 0.9. Given these parameters, and considering that the first two models have five independent variables and the third has six, the recommended sample size for each model was determined to be 73.

## Results

One hundred and twenty-four parent–child pairs were identified and invited to participate. Of these couples, 101 responded. The response rate was 81.5%, as indicated in a previous report with study flowchart [34]. All 101 participants remained during the 4-year follow-up period. Data on child attachment, average HbA1c, and HbA1c variability were collected for all patients (Table 1). Eight questionnaires assessing parental attachment were missing (ECR-RS, 7.8% of missing data), and we could calculate TIR for only 74 patients (26.7% of missing data), which affected further analyses. Baseline characteristics are shown in Table 1. Among the children, 50 (49.5%) were girls. Questionnaires were completed by mothers in 79.3%, fathers in 19.8%, and another caregiver (grandmother) in one case.

**Table 1:** Demographic data and descriptive comparison between sexes

	All	Females	Males	*p*
	*N* = 101	*N* = 50	*N* = 51	
Age in years	11.80 ± 2.09	12.04 ± 2.05	11.57 ± 2.13	0.236
Duration of T1D in years	5.31 ± 3.44	5.17 ± 3.23	5.45 ± 3.66	0.938
Hba1c average—% (mmol/mol)	8.03 (64) ± 0.96	8.10 (65) ± 1.07	7.97 (64) ± 0.84	0.770
Hba1c variability	0.075 ± 0.36	0.081 ± 0.04	0.070 ± 0.04	0.051
	*N* = 74	*N* = 35	*N* = 39	
TIR (% in 70–180 mg/dL)	48.78 ± 13.79	49.04 ± 13.38	48.45 ± 14.32	0.879
Average sensor glucose (CGM/BGM) (mg/dL)	183.30 ± 31.22	183.14 ± 33.94	183.44 ± 29.01	0.996
	*N* = 101	*N* = 50	*N* = 51	
Morning serum cortisol (ng/mL)	421.57 ± 147.03	410.65 ± 149.51	432.27 ± 145.30	0.472
CAI secure to at least one parent	65 (64.4%)	37 (74.0%)	28 (54.9%)	**0.045**
CAI secure to mother	63 (62.3%)	36 (72.0%)	27 (52.9%)	**0.044**
CAI insecure to mother	36 (35.6%)	13 (27.0%)	23 (36.1%)	
ECR-RS avoidance	*N* = 93 2.55 ± 0.94	*N* = 48 2.70 ± 1.04	*N* = 45 2.38 ± 0.79	0.089
ECR-RS anxiety	1.76 ± 0.90	1.73 ± 0.81	1.79 ± 0.99	0.895
Divorced family/living with one parent	*N* = 96 19 (19.8%)	*N* = 48 10 (20.8%)	*N* = 48 9 (18.8%)	0.798
Mother education level	*N* = 93	*N* = 48	*N* = 45	
Finished secondary school	48 (51.6%)	24 (51.1%)	24 (52.2%)	0.724
Finished university	22 (23.7%)	12 (25.5%)	10 (21.7%)	
Father education level	*N* = 93	*N* = 47	*N* = 46	
Finished secondary school	57 (61.3%)	29 (60.4%)	28 (62.2%)	0.866
Finished university	15 (16.1%)	8 (16.7%)	7 (15.6%)	
Mother employed	*N* = 94 80 (85.1%)	*N* = 47 39 (83.0%)	*N* = 47 41 (87.2%)	0.562
Father employed	*N* = 93 81 (87.1%)	*N* = 48 43 (89.6%)	*N* = 45 38 (84.49%)	0.460

Data are Mean ± *SD* or *N* (%)

*TIR* time in range, *CAI* Child Attachment Interview, *ECR-RS* Relationship Structures Questionnaire; Between-group comparisons: Student’s t-test or Pearson’s *χ*2 test, Mann–Whitney *U*-test, statistical significance *p* < 0.05 (bold)

Differences were found between girls and boys in attachment to the mother. A higher percentage of secure attachment was found in girls compared to boys (Pearson Chi-Square (1) = 4.014, *p* = 0.045, Table 1). Otherwise, there were no differences between the two groups.

The majority of children (83, 82.2%) used an insulin pump. There were no differences between users of different glucose monitoring modalities (*N*_CGM_ = 22, *N*_BGM_ = 52) in average Hb1Ac (Mann–Whitney *U* = 287, p = 0.61) or HbA1c variability (Mann–Whitney *U* = 212, *p* = 0.36). There were significant differences in TIR (*M*_CGM_ = 55.72, *M*_BGM_ = 45.84, *t* (38) = -2.90, *p* = 0.006), so glucose monitoring modality was included in further analyses. There were no differences in average Hb1Ac (*M-W U* = 810, *p* = 0.58), HbA1c variability (*M-W U* = 612, *p* = 0.23), or TIR (*t* (16) = -0.92, *p* = 0.37) between those using insulin pumps or mechanical injectors.

Average HbA1c was significantly related to HbA1c variability (*r* (99) = 0.59, *p* < 0.001) and TIR (*r* (72) = -0.41, p < 0.001), but there was no relationship between HbA1c variability and TIR (*r* (72) = −0.09, *p* = 0.43). Finally, diabetes duration was not statistically significantly related to average HbA1c (*r* (99) = 0.04, *p* = 0.71), HbA1c variability (*r* (99) = −0.09, *p* = 0.36), and TIR (*r* (72) = 0.02, *p* = 0.85) and was not included in further analyses.

Because concordance between children's attachment to mother and attachment to father was high (95% of children had the same two-way classification for both parents), we used only attachment to mother in further analyses to avoid multicollinearity between the independent variables. There was also a moderate correlation between parent attachment anxiety and parent attachment avoidance (*r* (91) = 0.43, *p* < 0.001), so two regression models (a and b) were created for each dependent variable.

### Predicting glycemic outcomes

When multivariable linear regression models were built to identify independent predictors of average Hb1Ac, HbA1c variability, and TIR, parental attachment anxiety (a) and attachment avoidance (b) were included alternately to avoid multicollinearity in the models. All final models achieved adequate fit. Statistical data for the models is presented in the Additional file 1: Table S2, Additional file 2: Table S3, and Additional file 3: Table S4, and statistically significant results are summarized in the Additional file 4: Table S5.

### Predictors of average HbA1c

**Fig. 1:**
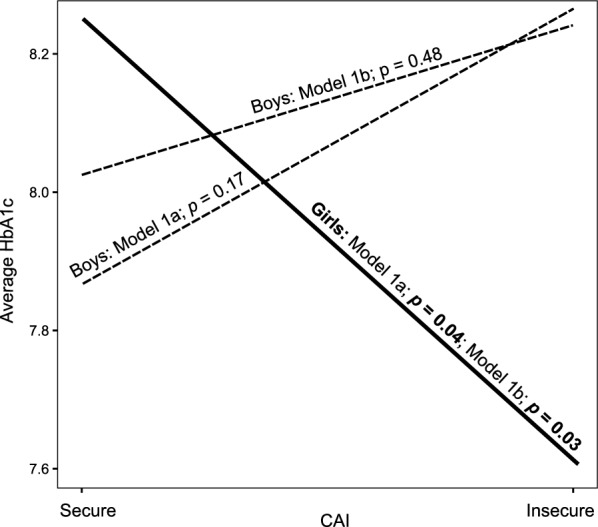
Simple slope analysis for the interaction between child sex and attachment from multivariable regression Models 1a and 1b. Statistically significant results appear in bold

In predicting the average HbA1c in the 4 years after inclusion Model 1a including ECR-RS anxiety achieved a global predictive power of *R*^2^ = 0.27, adjusted *R*^2^ = 0.18 (*F*(10, 78) = 2.981, *p* = 0.004) and Model 1b including ECR-RS avoidance had a global predictive power of *R*^2^ = 0.32, adjusted *R*^2^ = 0.24, *F*(10, 78) = 3.729, *p* < 0.001 (whole models are presented in Additional file 1: Table S2). Both models showed that a significant predictor of a higher average HbA1c was the older age of the child. The interaction between the child's attachment to the mother and the child's sex was statistically significant in both models. Simple slope analyses revealed that girls who were securely attached at baseline (CAI) had higher average HbA1c than girls who were insecurely attached (Model 1a: *B* = −0.61, *t* = −2.07, *p* = 0.04; Model 1b: *B* = −0.64, *t* = -2.25, *p* = 0.03, shown in Fig. 1). In boys, the opposite but statistically non-significant relationship was observed (Model 1a:* B* = 8.38, *t* = 1.39, *p* = 0.17).

**Fig. 2:**
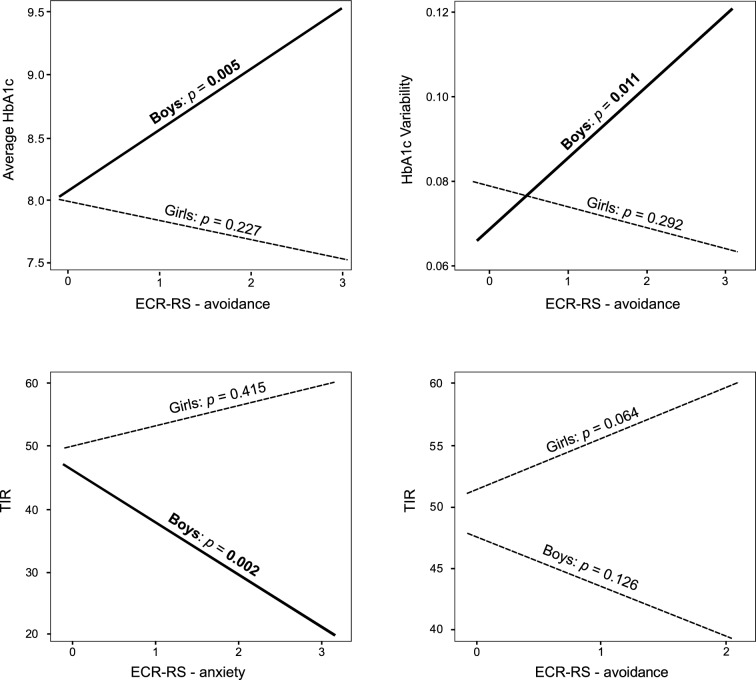
Simple slope analysis for statistically significant interaction between child sex and parent attachment style (ECR-RS anxiety or ECR-RS avoidance) from multivariable regression Models 1b, 2b, 3a and 3b. Statistically significant results appear in bold. Graphical results for Models 1a and 2a were not calculated because the interactions were not statistically significant

In Model 1b, higher parental attachment avoidance was associated with higher average HbA1c in boys (*B* = 0.51, *t* = 2.90,* p* = 0.005, shown in Fig. 2). For parents with high attachment avoidance (+ 1 *SD*), boys had higher average HbA1c than girls (*B* = 0.68, *t* = 2.34, *p* = 0.022), whereas for parents with low attachment avoidance (-1 *SD*), girls had higher average HbA1c than boys (*B* = −0.52,* t* = −2.02, *p* = 0.047).

### Predictors of HbA1c variability

The final models for predicting HbA1c Variability achieved adequate fit, Model 2a including ECR-RS anxiety with a global predictive power of *R*^2^ = 0.23, adjusted *R*^2^ = 0.16, *F*(8, 78) = 2.978 *p* = 0.006, and Model 2b including ECR-RS avoidance with a global predictive power of *R*^2^ = 0.28, adjusted *R*^2^ = 0.19, *F*(9, 77) = 3.299, p = 0.002 (whole models are presented in Additional file 2: Table S3). Both models showed that a significant predictor of higher HbA1c variability was older age of the child. In Model 2a, an independent predictor of higher HbA1c variability was also female sex.

Interactions in Model 2a showed higher HbA1c variability in children with the lowest morning serum cortisol levels (−1 SD) when their parents reported higher attachment anxiety (*B* = 0.013, *t* = 2.167, *p* = 0.033).

The following interactions statistically significantly predicted higher HbA1c variability in Model 2b: male sex in parents with higher attachment avoidance (*B* = 0.017,* t* = 2.595, *p* = 0.011, shown in Fig. 2), higher parental attachment avoidance in children with lowest cortisol levels (−1 *SD*) (*B* = 0.015, *t* = 2.750, p = 0.007) similar to Model 2a. In addition, in those whose parents reported low attachment avoidance (−1 *SD*) girls had higher HbA1c variability than boys (*B* = −0.031, *t* = −3.153, *p* = 0.002), and higher morning cortisol levels were associated with higher HbA1c variability (*B* = 7e-5, *t* = 2.144, *p* = 0.035).

### Predictors of TIR

Participants differed in TIR according to glucose monitoring modality, so CGM/BGM was included as an independent variable in the prediction of TIR. Both models achieved adequate fit, Model 3a including ECR-RS anxiety with a global predictive power of *R*^2^ = 0.41, adjusted *R*^2^ = 0.26, *F*(12, 50) = 2.846, *p* = 0.005, and Model 3b including ECR-RS avoidance with a global predictive power of *R*^2^ = 0.37, adjusted *R*^2^ = 0.22, *F*(12, 50) = 2.475 *p* = 0.013 (whole models are presented in Additional file 3: Table S4). In Model 3a, those who used BGM had a lower TIR than those who used CGM. Higher parental attachment anxiety predicted lower TIR in boys (*B* = −8.543, *t* = −3.231, *p* = 0.002; shown in Fig. 2) and in children with secure attachment to the mother (CAI) (*B* = −6.254, *t* = −2.094, *p* = 0.041). In children who have insecure attachment to their mothers (CAI), TIR decreased with increasing morning cortisol levels (*B* = −0.041, *t* = -2.193, *p* = 0.033). For children whose parents reported high attachment anxiety (+ 1 *SD*), girls had higher TIR than boys (*B* = −13.251,* t* = -2.451, *p* = 0.018).

Model 3b showed that higher child age significantly predicted lower TIR. Among participants with intermediate or higher (+ 1 *SD*) morning cortisol levels, use of CGM predicted higher TIR than use of BGM (intermediate cortisol: *B* = -9.895,* t* = −2.702, *p* = 0.009; + 1*SD* cortisol: *B* = −20.036, *t* = −3.336, p = 0.002). Among those whose parents reported high attachment avoidance (+ 1 *SD*), girls had higher TIR than boys (*B* = −11.350, *t* = −2.281, *p* = 0.027).

## Discussion

The aim of the present study was to investigate the influence of child attachment to mother, parental attachment style, and stress response, as measured by morning serum cortisol, on glycemic outcomes in children and adolescents with T1D. Multivariable linear regression models were used to predict average HbA1c, HbA1c variability, and TIR. By and large, the models independently showed that glycemic outcomes were predicted by age and interactions between other factors in most models, with sex and parental attachment style being the most important factors in the interactions.

Specifically, results indicated that girls were more likely to have secure attachment, which is consistent with results available in older children and adolescents [15, 40]. Results further indicated that children's attachment patterns were significantly associated with glycemic outcomes in girls, with securely attached girls having higher mean HbA1c than their insecure counterparts, contrary to expectations. The opposite was true for boys but did not reach statistical significance. Similar trends were reported by Costa Cordella et al. [31], who observed better glycemic outcomes (lower average HbA1c) associated with attachment security in boys but not in girls. The observed worse glycemic control in securely attached girls compared with insecurely attached girls may be due to the earlier transfer of responsibility for disease management from parents to girls in a more trusting parent–child relationship. Transfer of responsibility is widely recognized as a difficult process in diabetes care and an important factor associated with glycemic outcomes in older children and adolescents. The latter is also supported by findings that average HbA1c and HbA1c variability increase with age and TIR decreases. Similar findings of less favorable glycemic outcomes with increasing age have been shown by others [41].

In addition, results showed that in boys, parental attachment style was more strongly associated with glycemic outcomes than their own attachment style. Specifically, several regression models showed that the more insecure the parents' attachment, the higher the average Hb1Ac, HbA1c variability, and the lower TIR. Moreover, when parents reported lower levels of attachment avoidance (a characteristic of secure attachment in parents), girls had higher average Hb1Ac and HbA1c variability than boys. These findings are consistent with those reported by Costa-Cordella et al. in a sample of 77 children, in which mothers' attachment avoidance was positively correlated with their boys' HbA1c levels. Moreover, higher maternal attachment avoidance was associated with lower HbA1c levels in girls [31].

Parental attachment was also found to have a greater impact on diabetes outcomes in children with secure attachment (Model 3a) and in children with lower morning cortisol levels (Model 2a and 2b). These results are consistent with expectations that insecure attachment styles of parents would lead to worse blood glucose outcomes and that a less trusting relationship would lead to lower help-seeking behavior in children of insecure parents. Similar findings were reported by Costa-Cordella et al. in a cohort of 55 mother-son dyads, which were reflected in a negative correlation between maternal reflective functioning (an important feature of secure attachment that facilitates communication between parents and children) and HbA1c levels [42].

The role of cortisol appeared to be important in the group of insecurely attached children, as evidenced by a decrease in TIR with an increase in morning cortisol. The latter is in line with expectations, as a positive correlation between serum cortisol levels and HBA1c was previously observed [23]. In addition, attachment insecurity has been associated with cortisol responses [2, 15, 16]. The hypothesis was that children with a more intense stress response would find it more difficult to regulate their blood glucose levels, which would translate into poorer glycemic control. Cortisol can directly affect blood glucose fluctuations through its effect on the rise and fall of blood glucose levels, but the stressed child may also take poorer care of himself or herself. In particular, in cases where the parents showed low avoidance in their attachment style (secure form), a significant association was found between higher morning cortisol levels and higher HbA1c fluctuation. Thus, it could be concluded that children whose blood glucose fluctuates significantly show an increased stress response, which is more pronounced in secure parent–child relationships.

### Strengths and limitations

The present study was unique in that almost the entire cohort of children with T1D from Slovenia between the ages of 8 and 15 years was recruited. In addition, children's attachment to their parents was assessed using a well-established and validated interview measure, as opposed to a self-report measure that is often used in such studies. In addition, Hb1c levels were continuously tracked over a period of approximately 4 years. Moreover, a high proportion of children (82.2%) had an insulin pump, which is the standard of care for children with diabetes [43] and fully reimbursed by the national insurance company for all children and adolescents in Slovenia since 2002.

While these are considerable strengths, some limitations should also be noted. First, parental attachment was assessed using a self-report questionnaire, which, despite its reported reliability and validity [38], could be at risk of over- or under-reporting. Second, we know that other psychological factors, such as emotional or behavioral symptoms of the child or psychopathology of the parents, can influence blood glucose outcomes [44–46], so including assessment of psychopathology would improve the quality and generalizability of the results. Moreover, it was not possible to obtain TIR data from all participants. Finally, cortisol levels were measured only once, but we controlled for changes in circadian cortisol rhythms by collecting samples in the morning. Nonetheless, the results add to the growing literature and highlight the need for further research to identify and explain significant factors that may influence glycemic outcomes across development.

Overall, our research confirms that the influence of psychological factors on diabetes management is relationship- and sex-specific. Our study also raises questions for further research. It would be important to further define the role of cortisol and other stress hormones and psychopathology in children with T1D and their parents in relation to disease management. Given the growing literature on the relationship between attachment and disease progression, it would be imperative to begin evaluating evidence-based attachment-oriented interventions in this population. With the development of new technologies, better quality of diabetes management data is already possible, which will contribute to even more reliable research.

## Conclusion

In summary, the current findings suggest that attachment is closely related to glycemic outcomes in children with T1D, suggesting important sex differences. Consequently, attachment needs to be included as a factor in the regular assessment and management of girls and boys with T1D. Clinicians should pay particular attention to those girls who are independent and compliant (indicating secure attachment) but not yet able to control their diabetes. Although it may not be obvious, a gradual transition of responsibility for disease management is needed for them as well, one that does not release them into responsibility too early and thus ensures adequate support over time. On the other hand, boys whose parents express more insecurity in their relationships should also receive additional attention. Such families might benefit from additional counselling or family therapy.

With an increasing proportion of children using artificial intelligence for diabetes management, perhaps the influence of psychological factors could be significantly reduced in the future without other interventions. This remains to be determined, but in the meantime, our goal should be to achieve the best possible care with available interventions and to provide evidence for their use.

### Supplementary Information

**Additional file 1: Table S2.** Multivariable linear regression models reporting predictors of Average HbA1c.

**Additional file 2: Table S3.** Multivariable linear regression models reporting predictors of HbA1c variability.

**Additional file 3: Table S4.** Multivariable linear regression models reporting predictors of TIR.

**Additional file 4: Table S5.** Regression models to predict glycemic outcomes—overview of significant results.

## Data Availability

The data that support the findings of this study are not publicly available due to data protection regulations but are available from the corresponding author (S.K.) upon reasonable request.

## Abbreviations

HbA1c: Glycated hemoglobin
TIR: Time in range
T1D: Type 1 diabetes
CAI: Child Attachment Interview
ECR-RS: Relationship Structures Questionnaire
HPA: Hypothalamic–pituitary–adrenal
CGM: Continuous glucose monitoring
BGM: Blood glucose monitor
AIC: Akaike Information Criterion

## References

[1] Buchberger B, Huppertz H, Krabbe L, Lux B, Mattivi JT, Siafarikas A (2016). Symptoms of depression and anxiety in youth with type 1 diabetes: a systematic review and meta-analysis. Psychoneuroendocrinology.

[2] Pascuzzo K, Cyr C, Moss E (2013). Longitudinal association between adolescent attachment, adult romantic attachment, and emotion regulation strategies. Attach Hum Dev.

[3] Pietromonaco PR, Powers SI (2015). Attachment and health-related physiological stress processes. Curr Opin Psychol.

[4] Attachment BJ (1997). Volume one of the attachment and loss trilogy.

[5] Waters E, Merrick S, Treboux D, Crowell J, Albersheim L (2000). Attachment security in infancy and early adulthood: a twenty-year longitudinal study. Child Dev.

[6] Schore AN (2002). Dysregulation of the right brain: a fundamental mechanism of traumatic attachment and the psychopathogenesis of posttraumatic stress disorder. Aust N Z J Psychiatry.

[7] Maunder RG, Hunter JJ (2009). Assessing patterns of adult attachment in medical patients. Gen Hosp Psychiatry.

[8] Ciechanowski PS, Hirsch IB, Katon WJ (2002). Interpersonal predictors of HbA(1c) in patients with type 1 diabetes. Diabetes Care.

[9] Sund AM, Wichstrøm L (2002). Insecure attachment as a risk factor for future depressive symptoms in early adolescence. J Am Acad Child Adolesc Psychiatry.

[10] Sepa A, Ludvigsson J (2006). Psychological stress and the risk of diabetes-related autoimmunity: a review article. NeuroImmunoModulation.

[11] Rosenberg T, Shields CG (2009). The role of parent-adolescent attachment in the glycemic control of adolescents with type 1 diabetes: a pilot study. Fam Syst Health.

[12] Allen JP, McElhaney KB, Land DJ, Kuperminc GP, Moore CW, O'Beirne-Kelly H (2003). A secure base in adolescence: markers of attachment security in the mother-adolescent relationship. Child Dev.

[13] Miller GE, Chen E, Parker KJ (2011). Psychological stress in childhood and susceptibility to the chronic diseases of aging: moving toward a model of behavioral and biological mechanisms. Psychol Bull.

[14] MacMillan HL, Georgiades K, Duku EK, Shea A, Steiner M, Niec A (2009). Cortisol response to stress in female youths exposed to childhood maltreatment: results of the youth mood project. Biol Psychiatry.

[15] Borelli JL, Crowley MJ, David DH, Sbarra DA, Anderson GM, Mayes LC (2010). Attachment and emotion in school-aged children. Emotion.

[16] Luijk MP, Saridjan N, Tharner A, van Ijzendoorn MH, Bakermans-Kranenburg MJ, Jaddoe VW (2010). Attachment, depression, and cortisol: deviant patterns in insecure-resistant and disorganized infants. Dev Psychobiol.

[17] Korczak DJ, Madigan S, Manassis K, Daneman D (2016). The association of cortisol stress response with early adversity and diabetes control in adolescents with diabetes. J Depression Anxiety.

[18] Charmandari E, Achermann JC, Carel JC, Soder O, Chrousos GP (2012). Stress response and child health. Sci Signal.

[19] Dierckx B, Dieleman G, Tulen JH, Treffers PD, Utens EM, Verhulst FC (2012). Persistence of anxiety disorders and concomitant changes in cortisol. J Anxiety Disord.

[20] Carlsson E, Frostell A, Ludvigsson J, Faresjö M (2014). Psychological stress in children may alter the immune response. J Immunol.

[21] Sharif K, Watad A, Coplan L, Amital H, Shoenfeld Y, Afek A (2018). Psychological stress and type 1 diabetes mellitus: what is the link?. Expert Rev Clin Immunol.

[22] Gaete X, Iñiguez G, Linares J, Avila A, Mericq V (2013). Cortisol hyporesponsiveness to the low dose ACTH test is a frequent finding in a pediatric population with type 1 diabetes mellitus. Pediatr Diabetes.

[23] Ortiz R, Kluwe B, Odei JB, Echouffo Tcheugui JB, Sims M, Kalyani RR (2019). The association of morning serum cortisol with glucose metabolism and diabetes: the Jackson heart study. Psychoneuroendocrinology.

[24] Zhou JJ, Nuyujukian DS, Reaven PD (2021). New insights into the role of visit-to-visit glycemic variability and blood pressure variability in cardiovascular disease risk. Curr Cardiol Rep.

[25] Battelino T, Danne T, Bergenstal RM, Amiel SA, Beck R, Biester T (2019). Clinical targets for continuous glucose monitoring data interpretation: recommendations from the international consensus on time in range. Diabetes Care.

[26] Vigersky RA, McMahon C (2019). The relationship of hemoglobin A1C to time-in-range in patients with diabetes. Diabetes Technol Ther.

[27] Forbes A, Murrells T, Mulnier H, Sinclair AJ (2018). Mean HbA(1c), HbA(1c) variability, and mortality in people with diabetes aged 70 years and older: a retrospective cohort study. Lancet Diabetes Endocrinol.

[28] Critchley JA, Carey IM, Harris T, DeWilde S, Cook DG (2019). Variability in glycated hemoglobin and risk of poor outcomes among people with type 2 diabetes in a large primary care cohort study. Diabetes Care.

[29] Laffel LM, Kanapka LG, Beck RW, Bergamo K, Clements MA, Criego A (2020). Effect of continuous glucose monitoring on glycemic control in adolescents and young adults with type 1 diabetes: a randomized clinical trial. JAMA.

[30] Beck RW, Calhoun P, Kollman C (2012). Use of continuous glucose monitoring as an outcome measure in clinical trials. Diabetes Technol Ther.

[31] Costa-Cordella S, Luyten P, Giraudo F, Mena F, Shmueli-Goetz Y, Fonagy P (2020). Attachment and stress in children with type 1 diabetes and their mothers. Rev Chil Pediatr.

[32] Shayeghian Z, Moeineslam M, Hajati E, Karimi M, Amirshekari G, Amiri P (2020). The relation of alexithymia and attachment with type 1 diabetes management in adolescents: a gender-specific analysis. BMC Psychology.

[33] Bizzi F, Della Vedova AM, Prandi E, Cavanna D, Manfredi P (2021). Attachment representations to parents and emotional-behavioral problems: a comparison between children with type 1 diabetes mellitus and healthy children in middle childhood. Clin Child Psychol Psychiatry.

[34] Turin A, Dovč K, Klemenčič S, Bratina N, Battelino T, Lipovšek JK (2021). Carer's attachment anxiety, stressful life-events and the risk of childhood-onset type 1 diabetes. Front Psychiatry.

[35] Drobnič Radobuljac M, Shmueli-Goetz Y (2015). Attachment to caregivers and type 1 diabetes in children. Slovenian J Public Health.

[36] Vigers T, Chan CL, Snell-Bergeon J, Bjornstad P, Zeitler PS, Forlenza G (2019). cgmanalysis: an R package for descriptive analysis of continuous glucose monitor data. PLoS ONE.

[37] Shmueli-Goetz Y, Target M, Fonagy P, Datta A (2008). The child attachment interview: a psychometric study of reliability and discriminant validity. Dev Psychol.

[38] Rocha G, Peixoto E, Primi T, Motta I, Wiethaeuper D (2017). The experiences in close relationships - relationship structures questionnaire (ECR-RS): validity evidence and reliability. Psico-USF.

[39] Zhang Z (2016). Variable selection with stepwise and best subset approaches. Ann Transl Med.

[40] Gloger-Tippelt G, Kappler G (2016). Narratives of attachment in middle childhood: do gender, age, and risk-status matter for the quality of attachment?. Attach Hum Dev.

[41] Wood JR, Miller KM, Maahs DM, Beck RW, DiMeglio LA, Libman IM (2013). Most youth with type 1 diabetes in the T1D exchange clinic registry do not meet American diabetes association or international society for pediatric and adolescent diabetes clinical guidelines. Diabetes Care.

[42] Costa-Cordella S, Luyten P, Cohen D, Mena F, Fonagy P (2021). Mentalizing in mothers and children with type 1 diabetes. Dev Psychopathol.

[43] Dovc K, Telic SS, Lusa L, Bratanic N, Zerjav-Tansek M, Kotnik P (2014). Improved metabolic control in pediatric patients with type 1 diabetes: a nationwide prospective 12-year time trends analysis. Diabetes Technol Ther.

[44] Rechenberg K, Whittemore R, Grey M (2017). Anxiety in youth with type 1 diabetes. J Pediatr Nurs.

[45] Hood KK, Rausch JR, Dolan LM (2011). Depressive symptoms predict change in glycemic control in adolescents with type 1 diabetes: rates, magnitude, and moderators of change. Pediatr Diabetes.

[46] Eckshtain D, Ellis DA, Kolmodin K, Naar-King S (2010). The effects of parental depression and parenting practices on depressive symptoms and metabolic control in urban youth with insulin dependent diabetes. J Pediatr Psychol.

