# Supplementary material for: Attachment in close relationships and glycemic outcomes in children with type 1 diabetes
Source: Child Adolesc Psychiatry Ment Health. 2023 Oct 17;17:121. doi: 10.1186/s13034-023-00672-1 (PMC10583356; doi:10.1186/s13034-023-00672-1)
Supplement: Supplementary file 1 — Additional file 1: Table S2. Multivariable linear regression models reporting predictors of Average HbA1c. [file 13034_2023_672_MOESM1_ESM.doc]

**Table 2.** Multivariable linear regression models reporting predictors of Average HbA1c.

| **Predictors** | ***B*** |  | ***SE*** | ***t*** | ***p*** |
| --- | --- | --- | --- | --- | --- |
| Model 1a (Intercept) | 8.106 |  | 0.154 | 52.583 | < .001 |
| **CAI** | **-0.612** |  | **0.296** | **-2.067** | **0.042** |
| Sex | -0.228 |  | 0.247 | -0.884 | 0.380 |
| ECR-RS anxiety | -0.179 |  | 0.165 | -1.083 | 0.282 |
| Cortisol | 0.001 |  | 0.001 | 1.771 | 0.080 |
| **Age** | **0.201** |  | **0.067** | **2.985** | **0.004** |
| **CAI * Sex** | **0.997** |  | **0.405** | **2.462** | **0.016** |
| Sex * ECR-RS anxiety | 0.402 |  | 0.220 | 1.820 | 0.073 |
| Sex * Cortisol | -0.002 |  | 0.001 | -1.716 | 0.090 |
| Sex * Age | -0.152 |  | 0.095 | -1.592 | 0.115 |
| Cortisol * Age | < 0.001 |  | < 0.001 | 1.476 | 0.144 |
|  |  |  |  |  |  |
| Model 1b (Intercept) | 8.121 |  | 0.150 | 54.123 | <0.001 |
| **CAI** | **-0.642** |  | **0.285** | **-2.25** | **0.027** |
| Sex | -0.063 |  | 0.248 | -0.254 | 0.800 |
| ECR-RS aviodance | -0.139 |  | 0.127 | -1.094 | 0.277 |
| Cortisol | 0.001 |  | 0.001 | 1.744 | 0.085 |
| **Age** | **0.200** |  | **0.066** | **3.013** | **0.003** |
| **CAI * Sex** | **0.837** |  | **0.398** | **2.105** | **0.039** |
| **Sex * ECR-RS avoidance** | **0.650** |  | **0.216** | **3.001** | **0.004** |
| Sex * Cortisol | -0.002 |  | 0.001 | -1.574 | 0.120 |
| Sex * Age | -0.172 |  | 0.092 | -1.874 | 0.065 |
| Cortisol * Age | 0.001 |  | 0.000 | 1.847 | 0.068 |

CAI – Child Attachment to Mother, two-way classification (CAI), ECR-RS anxiety - Parent’s Attachment Anxiety (ECR-RS), ECR-RS avoidance - Parent’s Attachment Avoidance (ECR-RS), Cortisol - Morning Serum Cortisol, *B* - regression coefficient, *SE* - standard error of coefficient*, t* – *t*-value, *p* - level of statistical significance. Values of variables considered statically significant appear in bold text.
