# Supplementary material for: Attachment in close relationships and glycemic outcomes in children with type 1 diabetes
Source: Child Adolesc Psychiatry Ment Health. 2023 Oct 17;17:121. doi: 10.1186/s13034-023-00672-1 (PMC10583356; doi:10.1186/s13034-023-00672-1)
Supplement: Supplementary file 2 — Additional file 2: Table S3. Multivariable linear regression models reporting predictors of HbA1c variability. [file 13034_2023_672_MOESM2_ESM.doc]

**Table 3.** Multivariable linear regression models reporting predictors of HbA1c variability.

| **Predictors** | ***B*** | ***SE*** | ***t*** | ***p*** |
| --- | --- | --- | --- | --- |
| Model 2a (Intercept) | 8.397e-2 | 5.738e-3 | 14.635 | < .001 |
| CAI | -1.229e-2 | 1.113e-2 | -1.105 | 0.273 |
| **Sex** | **-2.101e-2** | **8.873e-3** | **-2.368** | **0.020** |
| ECR-RS anxiety | -4.049e-3 | 6.091e-3 | -0.665 | 0.508 |
| Cortisol | 7.439e-6 | 2.498e-5 | 0,298 | 0.767 |
| **Age** | **5.281e-3** | **1.771e-3** | **2.982** | **0.004** |
| CAI * Sex | 2.833e-2 | 1.521e-2 | 1.862 | 0.066 |
| Sex * ECR-RS anxiety | 1.559e-2 | 8.304e-3 | 1.878 | 0.064 |
| **ECR-RS anxiety * Cortisol** | **-6.481e-5** | **3.008e-5** | **-2.155** | **0.034** |
|  |  |  |  |  |
| Model 2b (Intercept) | 8.496e-2 | 5.614e-3 | 15.133 | < .001 |
| CAI | -1.646e-2 | 1.086e-2 | -1.515 | 0.134 |
| **Sex** | **-1.841e-2** | **9.090e-3** | **-2.026** | **0.046** |
| ECR-RS aviodance | -9.269e-3 | 5.711e-3 | -1.623 | 0.109 |
| Cortisol | 1.150e-5 | 2.456e-5 | 0.468 | 0.641 |
| **Age** | **4.638e-3** | **1.727e-3** | **2.686** | **0.009** |
| CAI * Sex | 2.342e-2 | 1.523e-2 | 1.537 | 0.128 |
| CAI * ECR-RS aviodance | 1.103e-2 | 8.139e-3 | 1.355 | 0.179 |
| **Sex * ECR-RS aviodance** | **2.213e-2** | **8.128e-3** | **2.722** | **0.008** |
| **ECR-RS avoidance * Cortisol** | **-6.492e-5** | **2.959e-5** | **-2.194** | **0.031** |

CAI – Child Attachment to Mother, two-way classification (CAI), ECR-RS anxiety – Parent’s Attachment Anxiety (ECR-RS), ECR-RS avoidance – Parent’s Attachment Avoidance (ECR-RS), Cortisol - Morning Serum Cortisol, *B* - regression coefficient, *SE* - standard error of coefficient*, t* – *t*-value, *p* - level of statistical significance. Values of variables considered statically significant appear in bold text.
