# Supplementary material for: Attachment in close relationships and glycemic outcomes in children with type 1 diabetes
Source: Child Adolesc Psychiatry Ment Health. 2023 Oct 17;17:121. doi: 10.1186/s13034-023-00672-1 (PMC10583356; doi:10.1186/s13034-023-00672-1)
Supplement: Supplementary file 3 — Additional file 3: Table S4. Multivariable linear regression models reporting predictors of TIR. [file 13034_2023_672_MOESM3_ESM.doc]

**Table 4.** Multivariable linear regression models reporting predictors of TIR.

| **Predictors** | ***B*** | ***SE*** | ***t*** | ***p*** |
| --- | --- | --- | --- | --- |
| Model 3a (Intercept) | 45.888 | 2.983 | 15.386 | <0.001 |
| CAI | 0.271 | 3.411 | 0.079 | 0.937 |
| Sex | -1.305 | 3.903 | -0.334 | 0.740 |
| **CGM/BGM** | **13.334** | **4.910** | **2.716** | **0.009** |
| ECR-RS anxiety | -0.620 | 3.781 | -0.164 | 0.870 |
| Cortisol | -0.023 | 0.018 | -1.228 | 0.225 |
| Age | -1.076 | 0.803 | -1.341 | 0.186 |
| **CAI * ECR-RS anxiety** | **9.660** | **4.036** | **2.393** | **0.020** |
| **CAI * Cortisol** | **-0.054** | **0.023** | **-2.314** | **0.025** |
| Sex * CGM/BGM | -9.177 | 7.033 | -1.305 | 0.198 |
| **Sex * ECR-RS anxiety** | **-11.450** | **4.383** | **-2.612** | **0.012** |
| Sex * Cortisol | 0.042 | 0.022 | 1.895 | 0.064 |
| CGM/BGM * Cortisol | 0.051 | 0.026 | 1.914 | 0.061 |
|  |  |  |  |  |
|  |  |  |  |  |
| Model 3b (Intercept) | 57.047 | 3.637 | 15.683 | <0.001 |
| CAI | -0.771 | 3.451 | -0.223 | 0.824 |
| Sex | -3.711 | 3.328 | -1.115 | 0.270 |
| **CGM/BGM** | **8.486** | **3.788** | **2.240** | **0.029** |
| **ECR-RS aviodance** | **10.837** | **4.787** | **2.264** | **0.028** |
| **Cortisol** | **0.063** | **0.025** | **2.524** | **0.015** |
| **Age** | **-3.007** | **1.312** | **-2.291** | **0.026** |
| CAI * Cortisol | -0.036 | 0.024 | -1.509 | 0.138 |
| **Sex * ECR-RS avoidance** | **-9.585** | **4.009** | **-2.391** | **0.021** |
| CGM/BGM * ECR-RS avoidance | 9.494 | 4.943 | 1.921 | 0.060 |
| **CGM/BGM * Cortisol** | **0.068** | **0.029** | **2.382** | **0.021** |
| CGM/BGM * Age | -3.171 | 1.729 | -1.834 | 0.073 |
| Age * ECR-RS avoidance | -1.563 | 0.949 | -1.647 | 0.106 |

CAI – Child Attachment to Mother, two-way classification (CAI), ECR-RS anxiety – Parent’s Attachment Anxiety (ECR-RS), ECR-RS avoidance – Parent’s Attachment Avoidance (ECR-RS), Cortisol - Morning Serum Cortisol, *B* - regression coefficient, *SE* - standard error of coefficient*, t* – *t*-value, *p* - level of statistical significance. Values of variables considered statically significant appear in bold text.
