# Supplementary material for: Attachment in close relationships and glycemic outcomes in children with type 1 diabetes
Source: Child Adolesc Psychiatry Ment Health. 2023 Oct 17;17:121. doi: 10.1186/s13034-023-00672-1 (PMC10583356; doi:10.1186/s13034-023-00672-1)
Supplement: Supplementary file 4 — Additional file 4: Table S5. Regression models to predict glycemic outcomes—overview of significant results. [file 13034_2023_672_MOESM4_ESM.docx]

**Table 5.**  Regression models to predict glycemic outcomes - overview of significant results.

|  | | 1. **Average HbA1** | | 1. **HbA1c Variability** | | | 1. **Time in range (TIR)** | | |
| --- | --- | --- | --- | --- | --- | --- | --- | --- | --- |
| **Model** | | **1a** | adj *R*^2^ = 0.18, *p =* 0.004 | | **2a** | adj *R*^2^ = 0.16, *p =* 0.006 | **3a** | adj *R*^2^ = 0.26, *p* < 0.005 | |
| 1. **ECR-RS-anxiety** | **Age** | ↑ Age ► ↑Avg HbA1c | | ↑Age: ►↑ HbA1c variability | | | / | | |
|  | **Sex** | / | | / | | | **ANX +1*SD*:**  Females > Males TIR | | |
|  | **CAI** | **Females:**  Secure > Insecure Avg HbA1c | | / | | | / | | |
|  | **ECR-RS-anxiety** | / | | **Cortisol -1*SD*:**  ↑ANX► ↑HbA1c variability | | | **Males:**  ↑ANX ► ↓TIR  **Secure (CAI):**  ↑ANX ► ↓TIR | | |
|  | **Cortisol** | / | | / | | | **Insecure (CAI):**  ↑Cortisol► ↓TIR | | |
|  | **CGM/BGM*** |  | |  | | | CGM > BGM TIR | | |
|  |  |  | |  | | |  | | |
| **Model** | | **1b** | adj *R*^2^ = 0.24, *p* < 0.001 | **2b** | | adj *R*^2^ = 0.19, *p =* 0.002 | **3b** | | adj *R*^2^ = 0.22, *p =* 0.013 |
| 1. **ECR-RS-avoidance** | **Age** | ↑Age ► ↑Avg HbA1c | | ↑Age ► ↑HbA1c variability | | | ↑Age ► ↓TIR | | |
|  | **Sex** | **AVOID +1*SD*:**  Males > Females Avg HbA1c  **AVOID -1*SD*:**  Females > Males Avg HbA1c | | **AVOID -1*SD*:**  Females > Males HbA1c variability | | | **AVOID +1*SD*:**  Females > Males TIR | | |
|  | **CAI** | **Females:**  Secure > Insecure Avg HbA1c | | **/** | | | / | | |
|  | **ECR-RS-avoidance** | **Males:**  ↑AVOID ► ↑Avg HbA1c | | **Males:**  ↑AVOID ► ↑ HbA1c variability  **Cortisol -1*SD*:**  ↑AVOID ► ↑ HbA1c variability | | | / | | |
|  | **Cortisol** | / | | **AVOID -1*SD*:**  ↑Cortisol ► ↑ HbA1c variability | | | / | | |
|  | **CGM/BGM*** |  | |  | | | **Cortisol Mean /+1*SD*:**  CGM TIR > BGM TIR | | |

Outcome measures: 1. Avg HbA1c – Average HbA1c, 2. HbA1c Variability, 3. TIR – Time in range; Variables in all models: Age, Sex, Cortisol - Serum morning cortisol, CAI - attachment security to mother (secure/insecure), CGM/BGM* – glucose monitoring modality (TIR models only). Alternating variables: a. ANX - ECR-RS-anxiety, parent’s attachment anxiety, b. AVOID - ECR-RS-avoidance, parent’s attachment avoidance, *R*^2^ adj – adjusted regression coefficient, *p* - level of statistical significance. *SD* – standard deviation. / - no statistically significant outcomes.
